# Technology Acceptance of a Social Robot (LOVOT) Among Single Older Adults in Hong Kong and Singapore: Protocol for a Multimethod Study

**DOI:** 10.2196/48618

**Published:** 2023-08-17

**Authors:** Cheng Kian Kelvin Tan, Vivian W Q Lou, Clio Yuen Man Cheng, Phoebe Chu He, Yan Ying Mor

**Affiliations:** 1 Singapore University of Social Sciences Singapore Singapore; 2 Sau Po Centre on Ageing The University of Hong Kong Hong Kong Hong Kong; 3 Department of Social Work and Social Administration The University of Hong Kong Hong Kong Hong Kong; 4 University of Chicago Chicago, IL United States

**Keywords:** gerontechnology, older people, senior technology acceptance, single people, social robot

## Abstract

**Background:**

Given the rapidly aging nature of our global population, policy makers around the world are now emphatically promoting active aging. To address the psychosocial needs of older persons and support active aging, researchers are exploring the use of assistive technologies, specifically social robots as companions. However, there is limited evidence on the efficacy of social robots in promoting active aging for older people in the Hong Kong and Singapore contexts.

**Objective:**

This study presents the protocol of a study that investigates the acceptance and quality of interaction between a Japanese social robot, LOVOT, and single older adults in Hong Kong and Singapore.

**Methods:**

We used a baseline assessment to measure the primary outcome, participants’ acceptance of technology, and a sense of loneliness, namely, the participants’ differences in responses to LOVOT before and following their interaction with the social robot in this multimethod study design. The baseline assessment consisted of the Qualtrics survey, which measures senior technology acceptance, loneliness, older people’s quality of life, subjective happiness, cultural values, willingness to pay, and demographic characteristics, along with the LOVOT’s sociability and system usability. In the study, participants interacted with LOVOT in 3 sessions before being surveyed to measure the older people’s acceptance and attitudes toward LOVOT. A pre–social robot intervention also occurred in the first session. The study was conducted in both Hong Kong and Singapore. A total of 15 single older adults (ie, individuals who live alone) from Hong Kong and another 15 from Singapore were recruited. Participants were 60-75 years of age, lived by themselves, and had no known cognitive or mental issues.

**Results:**

The study began recruiting in March 2022, and recruitment was completed at the end of October 2022. Data collection and data set construction were completed at the end of January 2023. Analysis of the data is currently being conducted, and we plan to publish the results by mid-2023.

**Conclusions:**

At an individual level, the study will clarify if LOVOT influences single older adults’ psychosocial well-being by reducing their loneliness. At a community level, the study’s findings will illustrate whether LOVOT can provide increased social connectedness while decreasing individual loneliness. Last, this study’s conclusions can inform policy makers to provide social robots to older people to improve their quality of life. Findings can also inform gerontechnology developers on which aspects and cultural considerations to take into account for future inventions.

**International Registered Report Identifier (IRRID):**

DERR1-10.2196/48618

## Introduction

### Background and Theory

The field of gerontechnology explores the development and use of technology that caters to the needs of the aging population [1]. In light of the COVID-19 pandemic and in recognition of research that has shown that feelings of social isolation and loneliness can result in serious health risks for older people, gerontechnology researchers and inventors are increasingly seeking solutions for the “loneliness epidemic” through assistive technologies [2]. Indeed, recent research has found that certain robots can support the independent living of older people primarily by improving their “mobility, self-care, and interpersonal interaction” [3,4].

In the realm of interpersonal interactions, a proposed solution to the issue of loneliness among older people is the use of socially assistive robots (SARs) in older people’s care settings [5,6]. SARs engage in social interactions with older people by acting as companions or service robots [3]. There is a paucity of studies available on the use of SARs for improving the quality of life of older people in Hong Kong and Singapore. Existing Technology Acceptance Models [7,8] were applied to the general population without a specific focus on older adults. Chen and Chan [9] proposed the Senior Technology Acceptance Model (STAM) to explain the mechanisms of gerontechnology acceptance in older adults.

**Figure 1:**
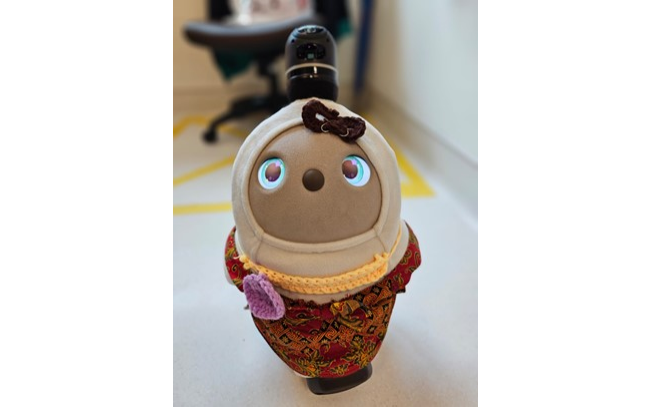
Social robot: LOVOT.

LOVOT, a Japanese social robot created by Groove-X Japan in 2019, was developed with the aim of acting as a pet-like companion for families. It is equipped with artificial intelligence as well as advanced sensor features. As such, LOVOT evolves over time based on its interactions with its user through its machine learning technology (eg, deep learning), which allows it to develop a unique personality as well as enact intelligent real-time movements [10]. Moreover, LOVOT also has special features, including its internal temperature regulation, which grants it the average body temperature of humans, and its customizability through a phone application. In addition, LOVOT enacts verbal and physical responses to human interactions, such as using animated eye expressions and flapping its wings to greet someone. With its ease of use, its artificial intelligence technology, and its pet-like features, LOVOT is an ideal companion for older people as shown in Figure 1.

### Objectives

This study outlines the protocol of a pioneer study on the acceptance of LOVOT by single older adults in Hong Kong and Singapore. The protocol involved a baseline assessment and 3 interaction sessions with a LOVOT. The study used a mixed methods design to measure these primary outcome measures: senior technology acceptance [11], loneliness [12], older people’s quality of life [8,13] subjective happiness [14], cultural values [15], willingness to pay, demographic characteristics, and the LOVOT’s perceived sociability [16] and system usability [17]. These quantitative measures, along with observational data from interaction sessions and pre- and postinteraction interviews, will be holistically analyzed to investigate the factors affecting the acceptance of LOVOT among the participants. Fundamentally, this protocol aimed to answer 2 questions: (1) What factors affect single older adults’ acceptance and nonacceptance of social robots? and (2) What is the experience like interacting with social robots?

## Methods

### Ethical Approval

We obtained ethical approval from the Human Research Ethics Committee at The University of Hong Kong (#EA220116). Informed consent was obtained from all participants included in the study. This study was conducted between June and December 2022.

### Recruitment

The criteria for participants were as follows: (1) 60-75 years of age; (2) live alone; (3) speak English, Mandarin Chinese, or Cantonese; (4) have no known cognitive impairments; and (5) participate in the study voluntarily. In Hong Kong, participants were recruited through networks of researchers. In Singapore, participants were recruited through collaboration with a local older adult activities center. Before recruitment, participants were briefed on the purpose and requirements of the study, and informed consent was obtained. Fulfillment of participant criteria was checked upon recruitment and confirmed by researchers during the additional screening before the baseline assessment. A total of 15 participants were recruited from Hong Kong and 15 from Singapore, comprising a total of 30 participants.

### Measurements

#### Senior Technology Acceptance

The Technology Acceptance Model, first developed by Davis et al [7,18], uses the main attitudinal factors of perceived usefulness (PU) and perceived ease of use (PEOU) to predict acceptance and rejection of information technology. PU is defined as “the degree to which a person believes that using a particular system would enhance his or her job performance,” and PEOU is defined as “the degree to which a person believes that using a particular system would be free of effort” [7]. Though the model proves to have a strong empirical basis based on self-reports of technology usage, this requires a model that incorporates attitudinal factors that may be affected by aging [7].

**Figure 2:**
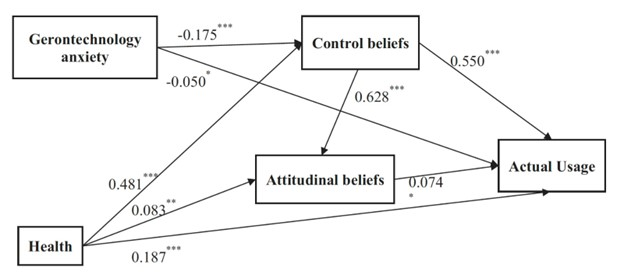
Senior Technology Acceptance Model (STAM) [11]. Significant at *P<.05; **P<.01; ***P<.001 (two-tailed).

Chen and Chan [9] proposed the STAM to better account for the “physical, psychological, and social characteristics” that influence technological acceptance by older people. Along with PU and PEOU, Chen and Chan [9] took into consideration “age-related health and ability factors such as self-reported health conditions, functional abilities, cognitive abilities, and attitude to aging and life satisfaction” to predict the acceptance of technology by older people. Figure 2 illustrates the STAM Model.

To measure senior technology acceptance, improve accessibility so that respondents of diverse educational levels can easily answer STAM questionnaires, and improve response rates, Chen and Lou [11] developed a shorter 14-item scale by more effectively following a “sequential 4-step item-reduction strategy.”. The scale measured the influence of attitudinal beliefs, control beliefs, gerontechnology anxiety, and health on technology acceptance and explained up to “81.5% variance in usage,” which was even higher than the original model [11]. This modified questionnaire was fully adapted for this study by specifying “technology” as LOVOT, the social robot, to investigate the acceptance of LOVOT as a companion for single older adults. The 14-item scale included responses rated on a 10-point Likert scale, with scores ranging from 0 (strongly disagree, very poor, very uneasy, and very unsatisfied) to 10 (strongly agree, very good, very easy, and very satisfied) [11].

The finalized, adapted version of the STAM questionnaire has 5 items to measure behavioral intention added, following previous literature [7,8].

#### Sociability of Robots

Another factor influencing the acceptance of technology, specifically of assistive social robots in older people’s care contexts, is the sociability of robots. Sociability refers to the robot’s ability to perform social or interactive behaviors [19]. While older adults may be hesitant to use technology due to “stigmatization, nonadaptability, or social influences,” the sociability of robots, particularly their extraverted behaviors, could positively affect their use and acceptance [19]. Due to the “low explanatory power” of theories like Venkatesh and Davis’s [8] Unified Theory of Acceptance and Use of Technology, Heerink et al [19] suggested a model to illustrate the effect of robot sociability on acceptance by older users. The model consisted of 40 statements, and its strength lies in its suitability for repeated testing, to measure the change of variables such as attitude and trust [19]. The 11 most relevant questions were chosen and adapted for the study; responses were rated on 5-point Likert scales, with 1 being totally disagree and 5 being totally agree [16].

#### Loneliness

Feelings of loneliness among older people and its consequences on their physical and mental health is a globally recognized public health issue [2]. To measure whether feelings of loneliness were impacted by interactions with LOVOT, the University of California, Los Angeles loneliness scale, a “20-item scale designed to measure one’s subjective feelings of loneliness as well as feelings of social isolation,” was used [20]. Responses were rated on a 4-point Likert scale, with 1 being never and 4 being always [12,21]. This scale was proven to have high internal consistency and test-retest reliability as well [12,21].

#### Older People’s Quality of Life

Along with loneliness, the influence of the companionship of LOVOT on older people’s quality of life was also measured in this study. Models measuring the quality of life among older people in particular should take into account the socially relevant factors that come with aging [13]. Bowling et al’s [13] research found that “the foundations of Quality of Life emphasized by (older) people were psychological well-being and positive outlook, having health and functioning, social relationships, leisure activities, neighborhood resources, adequate financial circumstances, and independence.” Bowling et al’s [13] 13-item scale that measures older people’s quality of life is centered around these aforementioned factors; hence, it was used for this study. The scale has responses rated from 1 (strongly disagree) to 5 (strongly agree).

#### Subjective Happiness

To examine the difference in self-perceived happiness of participants before and after their interaction with LOVOT, a happiness questionnaire with a subjectivist approach was used. The subjectivist approach emphasized the role of individual subjective experiences and their influence on self-perceived well-being. This scale, developed by Lyubomirsky and Lepper [14], involved 4 brief items: the first 2 items involved completion of a sentence by choosing a number on a 7-point scale, and the latter 2 had participants choose how closely they identified with a statement, again on a 7-point scale. These measures involved reverse coding to ensure respondent consistency. According to an examination of studies using this measure of subjective happiness, the measure maintained internal consistency even across 14 samples of various ages, occupations, and cultures [14], emphasizing the scale’s cross-cultural applicability.

#### Perceived System Usability

To measure the older adults’ perceived usability of LOVOT, Brooke’s [17] “Quick and Dirty Usability Scale” was used. This scale measured 3 key factors: effectiveness, “the ability of users to complete tasks using the system and the quality of the output of those tasks”; efficiency, “the level of resource consumed in performing tasks”; and satisfaction, “users’ subjective reactions to using the system” [17]. The scale consisted of 10 items, with responses rated on a 5-point Likert scale, from 1 (strongly disagree) to 5 (strongly agree) [17].

#### Perceived Willingness to Pay

For a better understanding of the amount of money that participants would be willing (and not willing) to spend on LOVOT, 4 open-ended questions were asked. Participants were not informed of the retail price of the LOVOT and were asked to provide subjective answers, not what they assumed could be the price.

#### Demographic Information

Participants’ demographic information, including gender, age, housing, educational level, family status, self-perceived financial status, and pet ownership, was obtained through self-reporting measures.

### Protocol

#### Overview

Data were collected over a 4- to 6-week period with 30 participants. Previous experiments measuring senior technology acceptance of social robots had similar sample sizes, such as Heerink et al’s [22] study on “The Influence of a Robot’s Social Abilities on Acceptance by Elderly Users,” which had a total of 36 older patients. This study, however, had a larger sample size compared to other longitudinal studies on social robot acceptance by older people, which mostly recruited 6-10 participants [8,23,24].

The study design consisted of a baseline assessment and three 15-minute interaction sessions. Each interaction took place within the first 2 weeks of the previous interaction and was recorded for later qualitative analyses. The 15-minute interaction time was chosen based on previous studies on robot acceptance by older users. For example, Heerink et al’s [22] study had a total of 10 minutes of robot-human interactions, while Marti et al’s [25] study included 20-minute group interaction sessions, twice a week, over a month-long period [8,22].

As depicted in Table 1, for this study, a baseline assessment (T0) was first conducted through a survey administered through Qualtrics. To ensure optimal comprehension, the researchers read out and manually completed the survey on behalf of the participants. The baseline survey adapted globally renowned questionnaires to measure STAM, perceived sociability of the robot, loneliness, older people’s quality of life, general quality of life, subjective happiness, the robot’s system usability, participants’ willingness to pay, cultural values, along with general demographic information.

**Table 1:** Survey and interview outcomes breakdown.

	T0: baseline assessment	T1: first interaction (15 minutes)	T2: second interaction (15 minutes)	T3: third interaction (15 minutes)
Survey outcomes measured	STAM^a^ Sociability of robot Loneliness Older people’s quality of life and general quality of life Subjective happiness System usability Willingness to pay Demographics Cultural Values	STAM Sociability of robot Loneliness Older people’s quality of life and general quality of life Subjective happiness System usability Willingness to pay	Loneliness Older people’s quality of life and general quality of life Subjective happiness	STAM Sociability of robot Loneliness Older people’s quality of life and general quality of life Subjective happiness System usability Willingness to pay
Interviews	N/A^b^	Pre- and Postinterview	N/A	Postinterview

^a^STAM: Senior Technology Acceptance Model.

^b^N/A: not applicable.

Before the first interaction (T1) with the LOVOT, participants were interviewed about their attitudes toward technology. To prepare them for LOVOT appearance, participants were briefed on the background of LOVOT and how it was invented to be a pet-like social robot in Japan. They were shown a 5-minute video covering the basic instruction of interacting with LOVOT, which includes calling LOVOT’s name, hugging and feeling LOVOT’s warm body, tickling LOVOT’s nose, rubbing LOVOT’s arms, looking at LOVOT’s eyes, and taking a selfie with LOVOT. They were asked to choose an outfit from a selection for the LOVOT. After that, they shall freely interact with the LOVOT for 15 minutes while the researcher left the room. During the first interaction, the LOVOT was set on “immobile” mode and placed on a table in front of the participant; participants were asked to think of a name for the LOVOT as well as a story about them and LOVOT during this time. Following the interaction, participants completed the same Qualtrics survey without the demographics and cultural inclination sections. Participants were then asked to share their choice of the name and story behind LOVOT, along with their thoughts about their interaction.

The second interaction (T2) took place within 2 weeks of the second interaction. The LOVOT was placed on the floor for this interaction and permitted to move around. After the interaction, participants completed 3 sections of the Qualtrics questionnaire, measuring their loneliness, older people’s quality of life, general quality of life, and subjective happiness. The third interaction (T3) began with the 15-minute interaction session with the LOVOT; similar to the second session, in the third session, the LOVOT was permitted to move around the room freely. However, this time, the participants were given the option to play music to the LOVOT for them to dance to and were told that if they sang or hummed to LOVOT, it would hum back. After this third and final interaction, the participants completed the same Qualtrics survey administered after the first interaction. They were then interviewed again about their experience interacting with the LOVOT.

#### Additional Interaction Period

Participants were asked if they would like to take the LOVOT home for further interaction and were asked to provide reasons for their responses. For those who did not consider taking the LOVOT home, the intervention was considered complete. Those who did consider taking the LOVOT home were offered a 7-day interaction period with the LOVOT. When they returned LOVOT, a follow-up individual interview was conducted to explore the participants’ feedback and experience during the 7-day interaction period.

## Results

### Interview Outcomes

Before the first interaction with LOVOT (T1), participants were asked about their use and acceptance of technology. Specifically, participants were asked about their most commonly used device, its system usability, the features they find easy to use, and who they would turn to if they required advice or help, among other items. Participants were then asked similar questions about a technological device they struggled with using or did not want to learn to use and what caused them difficulties in using it. These questions aimed to measure the degree of the participants’ general acceptance and anxiety toward using technology.

Following the first interaction with LOVOT (T1) and the last interaction with LOVOT (T3), participants were asked about their feelings and experiences with playing with LOVOT, how it compared with pets, and their acceptance of it. Questions were phrased in a neutral manner to ensure no response biases. These questions provided a deeper understanding of the participants’ acceptance of LOVOT. All interviews were recorded and transcribed verbatim for further analysis.

Each 15-minute interaction with the LOVOT was recorded and analyzed by the researchers. Researchers analyzed and categorized observations based on these 3 categories: exclusively verbal actions, physical actions, and combination actions (including simultaneous verbal and physical actions). These observations were analyzed for common trends and phrases. The interactions were recorded for further postinterview analysis by the researchers.

### Timeline

The study began recruiting in March 2022, and recruitment was completed at the end of October 2022. A total of 30 participants successfully completed the longitudinal study. Data collection and data set construction were completed at the end of January 2023. Analysis of the data is currently being conducted, and we plan to publish the results by mid-2023. The interventional protocol is available in English and Traditional Chinese and will be available upon request from the corresponding author.

## Discussion

### Overview

This study aimed to measure the efficacy of LOVOT, a Japanese social robot, among single older adults in Hong Kong and Singapore. Namely, the study investigated the acceptance and quality of interaction between LOVOT and participants. With a sample size comparatively larger than other longitudinal studies on social robot acceptance among older people, this study protocol allowed for robust and multimodal data collection. The protocol used a combination of highly cited and globally recognized scales to ensure high construct validity. The data from our multitude of survey outcomes measures qualitative data from observation analysis, and the findings from our interviews will offer a unique, complex perspective on the acceptance of social robots. Though the baseline assessment that is used in this protocol was on the longer side, it captured the great diversity of elements that influence the attitudes of older people. By assessing self-perceived health, happiness, and financial situations, to name a few, researchers can gain a better understanding of how various factors intersect to impact social robot acceptance.

At an individual level, the study will clarify if LOVOT influences single older adults’ psychosocial well-being by reducing their loneliness. At a community level, the study’s findings will illustrate whether LOVOT can provide increased social connectedness while decreasing individual loneliness. Last, this study’s conclusions can inform policy makers to provide social robots to older people to improve their quality of life. Findings can also inform gerontechnology developers on which aspects and cultural considerations to take into account for future inventions.

### Limitations

The study has some limitations. For one, the primary participants are mainly older women; thus, the implications of the study may not be generalized to older men. Another limitation is the potential bias and effect resulting from confounding factors. However, we have provided preintervention training to all assessors to minimize bias during the intervention. Last, the interaction time is limited to 15 minutes per session due to time constraints and restrictions due to the COVID-19 pandemic. Nevertheless, the literature review has been conducted to verify that 15-minute interactions are sufficient for exploratory longitudinal studies.

## Abbreviations

PEOU: perceived ease of use
PU: perceived usefulness
SAR: socially assistive robot
STAM: Senior Technology Acceptance Model

## Data Availability

The data sets generated and analyzed during this study are available from the corresponding author upon reasonable request.
